# Arthritis associated with Mycoplasma pneumoniae in a pediatric patient: A case report

**DOI:** 10.1097/MD.0000000000024316

**Published:** 2021-01-15

**Authors:** Cristina Oana Mărginean, Anca Meda Georgescu, Lorena Elena Meliţ

**Affiliations:** a Department of Pediatrics; b Department of Infectious Disease “George Emil Palade” University of Medicine, Pharmacy, Sciences and Technology from Târgu Mureş, Romania, Gheorghe Marinescu street no 38, Romania.

**Keywords:** arthritis, extrapulmonary manifestations, infant, Mycoplasma pneumoniae, pneumonia

## Abstract

**Introduction:**

*Mycoplasma pneumoniae* (MP) infection in infants is usually overlooked and it might result in important complications if left untreated. MP-induced arthritis is probably the least common extrapulmonary manifestation and frequently leads to delays in the diagnosis.

**Patient concerns:**

We report the case of a 2-year-old female child admitted in our clinic for prolonged fever (onset 2 weeks before the admission), for which the general practitioner established the diagnosis of acute pharyngitis and recommended antibiotics. But the fever persisted and the patient was referred to a pediatrician.

**Diagnosis:**

The laboratory tests revealed leukocytosis with neutrophilia, elevated C-reactive protein and liver cytolysis. The blood and urine cultures, as well as the serological hepatitis B and C, toxoplasmosis, Epstein Barr virus, Rubella, Herpes virus, and cytomegalovirus were negative. The chest X-ray established the diagnosis of pneumonia. The fever persisted for approximately 2 weeks after admission. On the 2nd week of admission, the patient began to experience gait difficulties complaining of pain in the right hip and ankle. The cardiology and pneumology consults revealed no pathological findings. The evolution was favorable after the initiation of Levofloxacin and MP infection was detected as we suspected. Moreover, the ultrasound of the hip revealed a mild joint effusion, while the ankle joint appeared to be normal at ultrasound. Thus, we established the diagnosis of hip and ankle arthritis based on the clinical and ultrasound findings.

**Interventions:**

Levofloxacin by vein was continued for 5 days, replaced afterwards with clarithromycin orally for 2 weeks.

**Outcomes:**

The gait difficulties persisted for approximately 5 months from the initial diagnosis, and improved once the titer of immunoglobulin M anti-MP antibodies lowered considerably. After more than 8 months, the patient was completely asymptomatic and the immunoglobulin M anti-MP was close to the normal range.

**Conclusion:**

The awareness of MP-induced arthritis in children represents the cornerstone in preventing diagnostic delays and initiating the proper treatment.

## 1 Introduction

*Mycoplasma pneumoniae* (MP), also known as an atypical bacterium, is 1 of the most frequent pathogens involved in the etiology of lower respiratory tract infections.^[1]^ Up to 40% of community-acquired pneumonia cases in children are due to this pathogen, being more prevalent in school-age children.^[2,3]^ Moreover, it seems that the prevalence of this infection differs also by geographic area because the reported cases in Taiwan and Japan described increased antibiotic resistance rate and complicated pneumonia accounting for up to 30% of intensive care unit admissions as compared to United States, where complicated cases were rarely reported.^[4,5]^ MP commonly occurs among children above the age of 6 years and it has been reported that in American children with community acquired-pneumonia requiring admission, MP infection was encountered in 19% above 6 years of age as compared to only 3% in those below this age.^[4,5]^ Thus, the scarcity of information regarding MP infection in children younger than 6 years of age is due to its rarity in this age group. The few reports on small children are contradictory since some of them underlined that younger children might express a worse evolution of this infection, while others revealed a milder course within this age range.^[6,7]^ The wide clinical spectrum of MP infection varies from asymptomatic defining a carrier state to severe cases of complicated pneumonia.^[8,9]^

Extrapulmonary manifestations represent another important face of MP infection since multiple organs were reported to be afflicted by this pathogen despite the multiple uncertainties related to the pathogenic mechanism. Nevertheless, 3 possible mechanisms are acknowledged: a direct type consisting of the essential role of inflammatory cytokines induced by the bacterium which is present at the inflammation site; an indirect type when the bacterium cannot be found at the site of inflammation, but it implies immune modulation consisting of autoimmunity or immune complexes formation expressing a major role; and vascular occlusion as the third possible pathogenic mechanism induced directly or indirectly by the bacterium.^[10–12]^ Most often these manifestations occur concomitantly with respiratory tract disease, but they were also reported as single manifestations.^[9]^ MP is a particular bacterium since its cell membrane contains only lipoproteins which seem to be involved in triggering the extrapulmonary manifestations.^[13]^ Arthritis is 1 of the least common extrapulmonary manifestations in children and therefore it is usually overlooked. The few reports on MP induced arthritis stated an incidence of this manifestation ranging between 2.1% and 0.9%.^[14,15]^ Moreover, the early detection of MP infection is essential in the evolution and prognosis of patients with extrapulmonary manifestations of MP taking into account that this infection is usually not considered in infants and antibiotics like macrolides are not commonly used. Thus, a delay in establishing a proper diagnosis along with the specific immunological immaturity for this age group might represent the main contributors of MP induced arthritis mechanism in infants.

The aim of this case report is to underline the diagnostic and management difficulties in a small child with arthritis due to MP infection.

The informed consent was obtained from the patient mother prior to the publication of this case report.

## 2 Case presentation

### 2.1 Presenting concerns

We report the case of a 2-year-old female child admitted in our clinic for prolonged fever (maximum 39°C) with the onset approximately 2 weeks before the admission, for which the general practitioner recommended antibiotic (Amoxicillin) establishing the diagnosis of acute pharyngitis, but the fever persisted and she was further referred to a pediatrician, who changed the antibiotic with Ceftriaxone. On the second day of administration, she developed a generalized rash and the limitations of head movements raising the suspicion of meningitis, which was ruled out by the specialist in infectious diseases, and the patient was admitted in our clinic for further investigations. Her history revealed only a preterm birth (35–36 gestational weeks).

### 2.2 Clinical findings

The clinical exam at the time of admission pointed out hyperemia of the pharynx, hypertrophy of the tonsils, with crypts, and limitations of active and passive movements of the head and neck, but no pain. The patient weighed 13 kg.

### 2.3 Diagnostic and therapeutic focus

The laboratory tests performed on the day of admission revealed leukocytosis (19,830 /μL), with neutrophilia (73.1%), elevated inflammatory biomarkers (C-reactive protein [CRP] 30 mg/L, erythrocyte sedimentation rate, 50 mm/h), liver cytolysis (aspartate amino-transferase 294.5 U/L). The peripheral blood analysis revealed only polymorphonuclear cells with toxic granulations. The blood and urine culture, as well as the serology tests for hepatitis B and C, toxoplasmosis, Epstein Barr virus, Rubella, Herpes virus, and cytomegalovirus were negative. A wide-spectrum double-regimen antibiotic therapy (Ceftriaxone + Amikacin) was initiated on the day of admission, but unfortunately the fever persisted and on the 5^th^ day of admission, the blood tests showed a mild increase of all laboratory parameters leukocytosis (21,220 /μL), neutrophilia (76.6%), and CRP 38.13 mg/L, but the limitations of neck and head movements disappeared. In spite of the normal respiratory sound at clinical exam, the chest radiography revealed an opacity in the right lung. Moreover, on the 2^nd^ week of admission the patient started to experience gait difficulties, complaining of hip and ankle pain and edema, with functional impotence of the inferior right limb. The cardiology consult revealed no pathological findings. Thus, we decided to change the antibiotics with Meropenem and Clindamycin based on the consult of the specialist in infectious diseases, who raised the suspicion of secondary infectious arthritis of the right knee. We also performed a pneumology consult, which ruled out possible tuberculosis. The fever spikes persisted for approximately 2 weeks after the admission each morning, but no more than 38°C. The leukocyte count remained above the normal limits as well as the CRP, which varied between 20 to 40 mg/L. We repeatedly assessed anti-nuclear antibodies, and they were initially elevated, but after approximately 2 weeks they became normal. Thus, we raised the suspicion of bacteriemia due to an infection with an atypical bacterium, and we introduced Levofloxacin with outstandingly favorable clinical course proved by the remission of fever. Nevertheless, gait difficulties persisted especially in the morning. Thus, we performed an inferior limb computed tomography, but it showed no pathological findings. Nevertheless, on the 5th day of Levofloxacin, she developed a severe hepatitis (aspartate aminotransferase 1713.8 U/L, alanine-aminotransferase, 1298.8 U/L). The parents decided to go to center specialized in infectious diseases, where an MP infection was detected (immunoglobulin M [IgM] anti-MP >200 U/ml, immunoglobulin G anti-MP 43.7 U/ml) as we already suspected, and clarithromycin orally was initiated for 2 weeks. Moreover, we repeated the hip and ankle ultrasound and we identified a mild effusion within the right hip joint. Thus, based on the clinical and ultrasound findings, we established the diagnosis of MP-related arthritis.

### 2.4 Follow-up and outcome

After approximately 1 month, the gait difficulties worsened and a rheumatology consult was performed, who recommended only non-steroid anti-inflammatory drugs. The titer of IgM anti-MP antibodies started to decrease (177.2 U/ml). Repeated ultrasound of the hip and knee revealed intraarticular fluid collection with spontaneous remission. The gait difficulties persisted for approximately 5 months from the initial diagnosis, they improved once the titer of IgM anti-MP antibodies lowered considerably (<80 U/mL). After more than 8 months, the patient is completely asymptomatic and the IgM anti-MP are almost within normal ranges (28 U/mL), whereas immunoglobulin G anti-MP increased considerably (>200 U/ml).

## 3 Discussions

MP infection is more common during winter as most respiratory pathogens and it may affect people of any age despite the fact that it is frequently reported in young people.^[14]^ Our patient was diagnosed with MP infection during winter. Multiple extrapulmonary manifestations are reported in patients with MP. Joint impairment in patients with MP infection is rare and 3 possible patterns are hypothesized: the first 1 is thought to appear during acute phase of this infection and it is migratory, polyarticular, affecting large joints;^[16]^ the second 1 follows the acute phase implying morning stiffness, hyperemia and joint edema of multiple medium-sized joints that might persist up to 1 year;^[17]^ while third is commonly seen in hypogammaglobulinemia patients in whom MP results in septic arthritis.^[18]^ The findings in our patient belong to the second pattern since she presented morning stiffness and joint edema that followed the acute phase and lasted approximately 5 months. Nevertheless, MP induced arthritis in children might fit also 1 of the other 2 pattern as in the case of a 7-year-old boy with hip impairment described by Ali et al.^[14]^ In a more recent study performed by Azumagawa et al, out of 348 patients diagnosed with MP infection, only 4 male children presented arthritis: 1 of 2 year-old, 2 of 4 years and 1 of 8 years.^[13]^ Nevertheless, our patient was a 2-year-old female. The 2-year-old boy described by Azumagawa et al presented with a history of fever and urticaria and developed right hip joint pain further migrating to the left hip and the ultrasound showed fluid collection around the hip joint.^[13]^ Similarly, our patient also presented with fever and rash before the admission. Moreover, the findings of Azumagawa et al suggest that the rash in our patient might have been misinterpreted as an allergic reaction to the antibiotic. Therefore, edema, erythema, pain and functional impotence must raise the suspicion of MP-related arthritis.^[15]^ The laboratory tests in the patients mentioned above revealed leukocytosis and a CRP of 29 mg/L, similar results to those encountered in our patient. Only 1 of the 4 patients described in the above-mentioned series, a 4-year-old boy presented pneumonia.^[13]^ In terms of our patient, in spite of no respiratory symptoms, the chest radiography revealed right pneumonia. Another study identified 13 cases with arthritis out of 1259 patients diagnosed with MP infection, but only 5 in children.^[15]^ These children diagnosed were between 1 and 7 years of age, and only 1 of them (a 1-year-old female) manifested impairment of multiple joints, the other 4 were monoarticular types. The fever in these pediatric patients lasted between 1 and 70 days, while arthritis lasted up to 4 weeks. In the case described by us, the fever lasted for approximately 1 month, while joint impairment almost 5 months. Hepatitis caused by MP infection is a rare manifestation and it was firstly described in1975.^[19]^ In the series of Ali et al., only 1 patient, a 55-year-old man presented hepatitis.^[14]^ This finding might be misinterpreted as allergic manifestation, poisoning,^[20]^ drug toxicity^[21]^ or as a secondary manifestations due to other conditions,^[22,23]^ before establishing the diagnosis of MP infection similar to our case who also presented liver dysfunction with a severe increase in transaminase levels during the clinical course, but whose evolution was outstandingly favorable. In spite of their doubtless importance, clinical findings must be confirmed by imagistic methods, such as ultrasound, computer tomography or even magnetic resonance imaging.^[13]^ Nevertheless, taking into account the migratory pattern, it is possible that imagistic changes could be missed during the routine examination. Thus, seriate ultrasound exams might be needed for detecting joint impairment.

Cardiovascular system involvement as a result of MP infection consists of intracardiac or intravascular thrombi, Kawasaki disease and, rarely, myocardial damage.^[24]^ MP seems to be associated with the presence of antiphospholipid antibodies in the human blood most likely due to a molecular mimicry between human phospholipids and bacterial components, this mechanism being incriminated in the development of thrombi.^[12]^ Dermatological manifestations associated to MP infection include erythema nodosum, cutaneous leukocytoclastic vasculitis, and rarely, Stevens-Johnson syndrome, Fuchs syndrome, toxic epidermal necrolysis or erythema multiforme major.^[12]^ The most common digestive manifestations due to this infection reported in the literature are liver dysfunction and necrotizing pancreatitis, while the neurological disorders consist of opsoclonus-myoclonus syndrome, striatal necrosis, disseminated encephalomyelitis, transient Parkinsonism, cerebellitis, acute cerebellar ataxia, and most recently described ‘mycoplasmal cerebral vasculopathy’.^[12]^ The last entity presents a slowly progressive clinical evolution consisting of episodic encephalopathy and movement disorders, being a true manifestation of MP infection since MP antigens were identified in the cytoplasm of cerebral microvascular endothelial cells and in microvascular lumina through histological studies.^[25]^ Other types of extrapulmonary manifestations involve hematopoietic system (splenic artery embolism), musculoskeletal system (rhabdomyolysis, arthritis), respiratory manifestations (pulmonary embolism) or urogenital ones (glomerulonephritis, glomerulonephritis with interstitial nephritis, renal artery embolism, pediatric priapism).^[12]^ Most of this information is provided in case reports due to their rarity and the relatively frequent rate of misdiagnosis. Taking into account that many of the aforementioned extrapulmonary manifestations occur in the absence of pneumonia, the most reliable diagnostic tool for these manifestations are serological methods. The treatment of extrapulmonary manifestations due to MP infection is complex involving immunomodulators like corticosteroids or immunoglobulins for the most severe cases, i.e. encephalitis or Stevens-Johnson syndrome; anticoagulant therapy manifestations implying vascular occlusion; and effective antibiotics against MP in order to reduce the excess of antigenic stimuli.^[12]^ Fortunately, no other extrapulmonary manifestations were identified in our case, except for arthritis, rash and hepatitis.

MP infection responds well to macrolides and fluoroquinolones, but these are not commonly used in pediatric patients, and thus a proper timely diagnosis is mandatory for a favorable evolution. Certain cases, especially those with severe extrapulmonary manifestations might benefit from corticosteroids administration. Of the 4 cases reported by Azumagawa et al, 3 were treated only with antibiotics, azithromycin or midecamycin, and one received betamethasone associated with azithromycin.^[13]^ Our patient received levofloxacin initially since we suspected a sepsis with an atypical bacterium, and as it is well-known that sepsis in infant might be a life-threatening condition.^[26]^ We further continued with clarithromycin orally once MP infection was serologically confirmed.

We can conclude that MP bacteremia can result in a wide-spectrum of extrapulmonary manifestations. MP infection in infants is usually overlooked and it might result in complications if left untreated. MP-induced arthritis is probably the least common extrapulmonary manifestation and frequently leads to delays in the diagnosis. Our case emphasizes once more the importance of MP related extrapulmonary manifestations in infants even in the absence of respiratory symptoms. The awareness of MP-induced arthritis in children represents the cornerstone in preventing diagnosis delays and initiating the proper treatment.

## Acknowledgments

”We express our sincere thanks to Prof Monica Luminos for performing her infectious disease expertise in this case.”

## Author contributions

MCO, MLE conceptualized and designed the study, drafted the initial manuscript, and reviewed and revised the manuscript.

MCO, MLE and GAM designed the data collection instruments, collected data, carried out the initial analyses, and reviewed and revised the manuscript.

All authors approved the final manuscript as submitted and agree to be accountable for all aspects of the work.

**Conceptualization:** Cristina Oana Mărginean, Lorena Elena Melit.

**Data curation:** Cristina Oana Mărginean.

**Formal analysis:** Anca Meda Georgescu.

**Investigation:** Cristina Oana Mărginean, Lorena Elena Melit.

**Methodology:** Cristina Oana Mărginean, Lorena Elena Melit.

**Resources:** Cristina Oana Mărginean.

**Supervision:** Cristina Oana Mărginean, Anca Meda Georgescu, Lorena Elena Melit.

**Validation:** Cristina Oana Mărginean, Anca Meda Georgescu.

**Visualization:** Cristina Oana Mărginean, Lorena Elena Melit.

**Writing – original draft:** Cristina Oana Mărginean, Lorena Elena Melit.

**Writing – review & editing:** Cristina Oana Mărginean, Lorena Elena Melit.

## Abbreviations

Abbreviations: CRP = C-reactive protein, IgM = immunoglobulin M, MP = mycoplasma pneumoniae.
